# Infection as a Differential Diagnosis of Solid Retroperitoneal Masses: A Case Series and Review of the Literature

**DOI:** 10.7759/cureus.24656

**Published:** 2022-05-01

**Authors:** Georgia Levidou, Tilman Klein, Kerstin Schaefer-Eckart, Clemens Huettenbrink, Panagiota Manava

**Affiliations:** 1 Department of Pathology, Nuremberg General Hospital, Paracelsus Medical University, Nuremberg, DEU; 2 Department of Urology, Nuremberg General Hospital, Paracelsus Medical University, Nuremberg, DEU; 3 Department of Oncology and Hematology, Nuremberg General Hospital, Paracelsus Medical University, Nuremberg, DEU; 4 Department of Radiology, Nuremberg General Hospital, Paracelsus Medical University, Nuremberg, DEU

**Keywords:** retroperitoneal tumour, bacillus calmette-guérin (bcg), differential diagnosi, histoplasma, aspergillus and candida, benign renal mass

## Abstract

The differential diagnosis of retroperitoneal masses includes a variety of benign and malignant conditions, among which infections constitute a significant subgroup. Familiarity with these infectious pseudotumours could facilitate prompt diagnosis. In this report, we describe three patients with an infectious pseudotumour, which was clinically and radiologically highly suggestive of a neoplasm.

The first patient was a 62-year-old woman with a history of Richter syndrome, who seven months after allogeneic haematopoetic stem cell transplantation from an unrelated donor presented with a renal mass. A renal biopsy at that time revealed necrotic tissue. The patient displayed multiple relapses of Richter syndrome (for which she received also chimeric antigen receptor T-cell therapy salvage chemotherapy) and remissions of the lymphoma as well as an Aspergillus pneumonia for which she was treated with intravenous ambisome and afterwards oral posaconazole. Since the renal mass persisted and to exclude malignancy, nephrectomy was performed which revealed the presence of fungal hyphae.

The second patient was a 51-year-old man with a history of a low-grade non-muscle-invasive bladder urothelial carcinoma, who after *Mycobacterium bovis *Calmette-Guerin instillation presentedwith fever and a suspicious renal mass. A partial nephrectomy was performed. Intraoperative frozen section analysis and routine histology suggested a *Mycobacterium bovis*-associated lesion, which was confirmed by polymerase chain reaction (PCR) analysis.

The third patient was an 85-year-old man who presented with loss of appetite, fatigue, and significant weight loss (24 Kg in less than a year) as well as a travel history. The laboratory tests showed a low sodium and a high potassium level. CT scans revealed a solitary lesion in the right lung, a small liver lesion as well as bilateral adrenal lesions. A CT-guided biopsy revealed the presence of *Histoplasma capsulatum, *which was confirmed by PCR analysis. A retrospective review of all parameters indicates that all three patients presented with some risk factors, such as immunosuppression, travel, or clinical history that could raise the suspicion of infection in order to be included in the differential diagnosis, thus providing an additional tool for timely diagnosis.

## Introduction

The differential diagnosis of a retroperitoneal renal or adrenal mass includes apart from solid neoplasms a variety of benign lesions, among which infectious diseases figure prominently. In some atypical cases especially in those caused by non-bacterial pathogens, the findings may be confusing, rendering the differential diagnosis other than malignancy challenging.

In this article, we report a case series of three patients with an infectious pseudotumour mimicking solid neoplasm (in two cases renal and in one case adrenal), review the respective literature, and attempt to retrospectively elucidate the surrounding evidence that could possibly lead to an earlier diagnosis in such cases. 

## Case presentation

Case 1

Clinical History

A 62-year-old woman was diagnosed initially with chronic lymphocytic leukaemia with 17p deletion and p53 mutation and received idelalisib and rituximab. Two years after initial treatment she displayed a transformation to a diffuse large B cell lymphoma (Richter syndrome). After seven cycles of R-CHOP (rituximab, cyclophosphamide, doxorubicin hydrochloride, vincristine, prednisolone) an allogeneic haematopoetic stem cell transplantation (aHSCT) from an unrelated human leukocyte antigen (HLA)-matched donor was performed. Immunosuppressive therapy consisted of methotrexate, cyclosporin, and anti-thymocyte globulin (ATG). The patient developed an acute cutaneous graft-versus-host disease (aGvHD) grade 3, for which she received prednisolone and ibrutinib resulting in complete regression. Nine months after HSCT, lymphoma relapsed again, and salvage therapy with R-DHAP (rituximab, dexamethasone, cytarabine, cisplatin) resulted in partial remission. Eleven months after HSCT, a chimeric antigen receptor (CAR) T-cell therapy was performed. Six months after CAR T-cell therapy, the lymphoma was in the third remission, but she developed a fungal pneumonia with multiple lesions in both lungs caused by *Aspergillus spp*., as confirmed by antigen-based analysis of the bronchoalveolar lavage fluid. Therapy with intravenous ambisome, and afterwards, oral posaconazole resulted in regression of the pulmonary infiltrates. Six months later, she was admitted to the hospital again with septic arthritis of the knee joint due to *Escherichia coli*, for which she received intravenous ceftazidime and afterwards oral ciprofloxacin. 

Laboratory Findings

At the time of the admission, the patient’s laboratory results showed anaemia (Hb 8.5 g/dl) leukopenia (3.7/nl), without neutropenia, and increased liver enzymes (alkaline phosphatase (ALP) 286U/l, gamma-glutamyl transferase (g-GT) 790U/l, aspartate amino-transaminase (AST) 93U/l, alanine amino-transaminase (ALT) 56U/l) (Table 1). Urinalysis showed increased leucocytes, erythrocyturia, and proteinuria but was negative for any microorganisms. 

**Table 1:** Case 1 - Laboratory findings on the day of admission.

Parameter	Lab value	Normal range
Leucocytes	3.7	4-10/nl
Erythrocytes	3.11	4.2-5.4/pl
Haemoglobin	8.5	12.0-16.0g/dl
Haematocrit	28.6	36-46%
Thrombocytes	245	140-440/nl
Sodium	145	136-145mmol/l
Potassium	4.2	3.4-4.5mmol/l
Urea	10	16.6-48.5mg/dl
Creatinine	0.47	0.5-0.9mg/dl
Alkaline Phosphatase	286	35-104mEq /l
g-GT (gamma-glutamyl transferase)	790	<40 mEq /l
AST (alanine transaminase)	93	<35mEq /l
ALT (aspartate aminotransferase)	56	<35mEq /l

Imaging Studies

**Figure 1:**
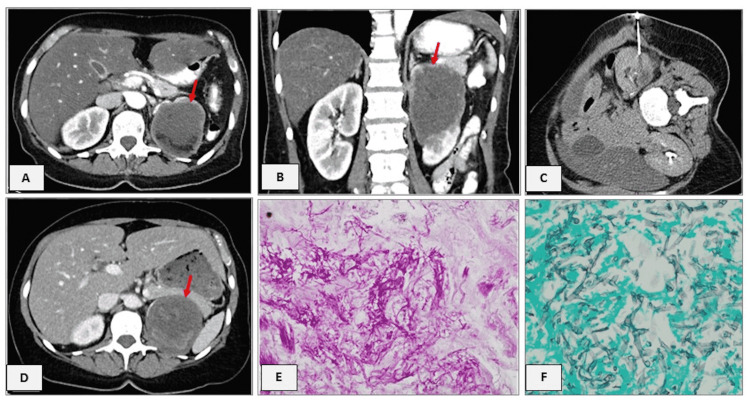
Case 1 - CT abdomen and histological findings. (A, B) Contrast-enhanced CT with a renal partly solid central inhomogeneous lesion (A and B: seven months after HSCT; D: preoperatively). (C) Axial CT image from biopsy procedure with a posterior oblique approach. Red arrows show the lesion. (D, E) Histological findings in the radical nephrectomy specimen showing amounts of fungal hyphae, suggesting according to the morphology *Aspergillus spp*, *Fusarium spp*. or *Pseudoallescheria boydii *(D. Hematoxylin-Eosin x100, E. Grocott-Gomori's methenamine silver stain x100) HSCT: haematopoetic stem cell transplantation

Seven months after HSCT, a CT scan of the abdomen showed a large 7.1 x 6.4 cm predominantly solid central inhomogeneous lesion with peripheral rim enhancement involving the renal parenchyma and the collecting system (Figure 1), which was constantly at the time of CAR T-cell therapy. Differential diagnosis included an abscess formation or a malignancy. CT-guided needle biopsy of the left kidney was performed, revealing the presence of necrotic tissue. Eighteen months later the patient was admitted with *Escherichia coli*-associated septic arthritis. At this time a new CT scan was performed, and the aforementioned mass was persistent. Because the lesion might have been the origin of the infectious episodes and to exclude malignancy with a tumor involving and replacing the majority of the renal parenchyma, a decision for a radical nephrectomy was made, in which the kidney and the surrounding fat tissue were removed.

Histological Findings and Diagnosis of Infection

The gross specimen revealed the presence of a 9.5 cm whitish-yellowish mass arising from the upper pole and affecting almost the entire renal parenchyma, leaving only a small rim of normal renal tissue in the lower pole, with communication to the renal pelvis, consisting of liquefied necrotic material. The renal pelvis mucosa was macroscopically unaffected. On microscopic examination, we found an encapsulated area with pronounced necrosis of the renal parenchyma which contained abundant amounts of fungal hyphae, whose differential diagnosis according to the morphology was *Aspergillus spp.*, F*usarium spp.,* and *Pseudoallescheria boydii *(Figure 1). The limited remaining viable renal parenchyma showed moderate interstitial nephritis and atrophied renal tubules. There was no evidence of B-cell lymphoma infiltrates. Since the lesion was already present at the time of the fungal pneumonia due to *Aspergillus spp.*, the diagnosis of an invasive aspergillosis was assumed.

Case 2

Clinical History

A 51-year-old man with a non-muscle-invasive pathological stage Ta (pTa) low-grade urothelial bladder carcinoma, diagnosed one year ago, for which due to multiple recurrences intravesical *Mycobacterium bovis *Calmette-Guerin (BCG) was initiated. After two courses of BCG therapy, an MRI of the abdomen was performed because of fever. 

Clinical and Laboratory Findings

The physical examination did not reveal any tenderness in the left kidney area. The laboratory tests showed an elevated glomerular filtration rate (GFR)(>60 mm/h) and mild anaemia (haematocrit: 37.2%, haemoglobin: 12.7 g/dl) (Table 2). Urinalysis revealed erythrocyturia but was negative for any microorganisms. 

**Table 2:** Case 2 - Laboratory findings on the day of admission.

Parameter	Lab value	Normal range
Leucocytes	9.2	4-10/nl
Erythrocytes	4.23	4.5-6.3/pl
Haemoglobin	12.7	14.0-18.0g/dl
Haematocrit	37.2	38-52%
Thrombocytes	204	140-440/nl
Sodium	136	136-145mmol/l
Potassium	2.12	3.4-4.5mmol/l
Urea	34	16.6-48.5mg/dl
Creatinine	0.92	0.5-0.9mg/dl

Imaging Studies 

**Figure 2:**
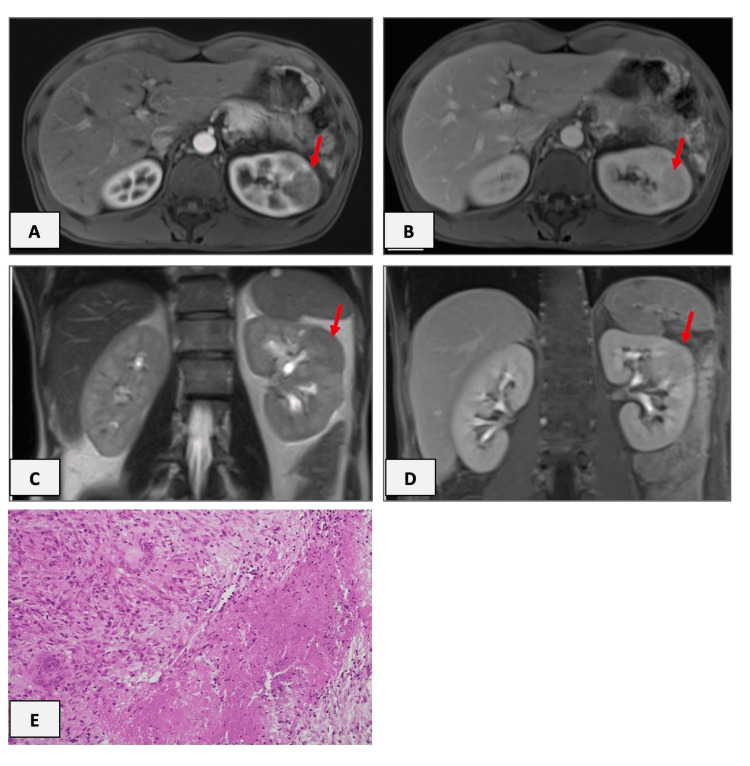
Case 2 - Magnetic resonance tomography scans and histological findings. Axial T1-weighted contrast-enhanced images showing a hypointense mass in the arterial phase (A) with slight enhancement in pv-phase (B). Coronal T2-w (C) and T1-w fat-sat. post-contrast images in the excretory phase (D) demonstrate a normal calyx (central unaffected calyx sign). Red arrows show the lesion.  (E) Histological findings showing a necrotizing granulomatous reaction which was considered compatible with a complication of the BCG instillation (Hematoxylin-Eosin x200). BCG: *Mycobacterium bovis* Calmette-Guerin

Magnetic resonance tomography (MRT) scan revealed a cortical mass on the left kidney measuring 3.4 x 3.4 cm, which in coronal T2-weighted images revealed to be a solid cortical mass. The lesion demonstrated low and delayed contrast enhancement. Coronal images of the excretory phase demonstrated a normal calyx (Figure 2). The lesion was considered to be highly suspicious of malignancy, and therefore the patient underwent partial nephrectomy and a 4 x 2 x 1.5 cm specimen was removed. 

Histological Findings and Diagnosis of Infection

An intraoperative frozen section analysis revealed a granulomatous lesion without any presence of malignant cells but with high suspicion of a mycobacteria-associated lesion. Permanent sections confirmed the presence of granulomatous pyelonephritis with centrally necrotising granulomas (Figure 2). Ziehl-Neelsen and Auramine O staining were negative for acid-fast bacteria. However, the histological appearance was suggested to be associated with BCG instillation. Direct polymerase chain reaction (PCR) performed in the kidney tissue identified *Mycobacterium tuberculosis* complex, whereas cultures in liquid medium identified *Mycobacterium bovis*. 

Therapy and Follow-Up

Further clinical examination did not reveal any other focus of the disease. The postoperative recovery of the patient was uneventful. According to the histologic diagnosis the patient received for six months a combination of three drugs (ethambutol, isoniazid, and rifampicin).

Six years thereafter, the patient is doing well without any signs of disease and no evidence of urothelial carcinoma identified in the follow-up.

Case 3

Clinical History

An 85-year-old man with a known moderate diastolic heart failure (New York Heart Association (NYHA) Class III) as well as mitral and tricuspid valve regurgitation grade I was admitted to our hospital, due to progressive shortness of breath, body weakness, and deterioration of his general condition as well as frequent anxiety attacks. The patient presented with loss of appetite, and easy fatigue and reported a loss of 24 Kg of his total body weight in less than a year. The patient had a significant travel history in the past years to India and therefore there was a high suspicion of infection. A transoesophageal echocardiogram was performed in order to rule out endocarditis. A bronchoscopy was performed but was negative for any lesion or inflammatory alterations. 

Clinical and Laboratory Findings

On physical examination, he had a pulse rate of 84/min and low blood pressure. The laboratory tests showed a low sodium (129 mEq/L) and a high potassium (5.3 mEq/L) level (Table 3). The adrenocorticotrophic hormone (ACTH) level was elevated, and the ACTH stimulation test was normal. The serum norepinephrine level was also elevated (2187 ng/dl). Serum metanephrine and normetanephrine levels were however normal. The bronchoalveolar lavage fluid analysis, in which a hematoxylin-eosin (HE) and a periodic acid-Schiff (PAS) stain were performed, was also negative for tumour cells and contained a few inflammatory cells, such as neutrophils. Multiple blood culture analyses were negative for aerobic or anaerobic bacteria.

**Table 3:** Case 3 - Laboratory findings on the day of admission.

Parameter	Lab value	Normal range
Leucocytes	10.4	4-10/nl
Erythrocytes	5.94	4.5-6.3/pl
Haemoglobin	16.7	14.0-18.0g/dl
Haematocrit	45.4	38-52%
Thrombocytes	190	140-440/nl
Sodium	129	136-145mmol/l
Potassium	5.3	3.4-4.5mmol/l
Urea	60	16.6-48.5mg/dl
Creatinine	1.87	0.5-0.9mg/dl
Alkaline Phosphatase	77	35-104mEq /l
g-GT (gamma-glutamyl transferase)	38	<40 mEq /l
AST (alanine transaminase)	22	<35mEq /l
ALT (aspartate aminotransferase)	14	<35mEq /l
ACTH (adrenocorticotrophic hormone)	163	7.2-63.3pg/ml
Cortisol	14.9	6.02-18.4µg/dl
Norepinephrine	2187	70-1700ng/l
Normetanephrine	28	18-111 pg/mL
Metanephrine	14	12-60 pg/mL

Imaging Studies

**Figure 3:**
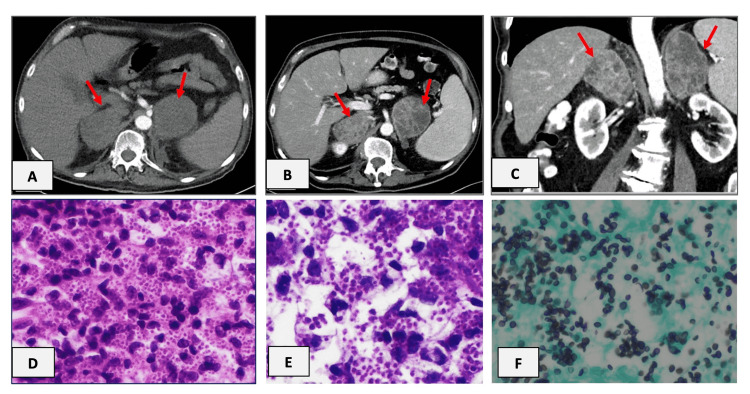
Case 3 - CT chest and abdomen and histological findings. Contrast-enhanced CT (arterial and portal venous phase) with bilateral adrenal enlargement. Non-enhancing adrenal masses in arterial scan (A). Portal-venous scan (B, C) demonstrates an inhomogeneous enhancement with hypointense, regressive areas. Red arrows show the adrenal enlargement.  (D-F) Histological findings of the adrenal biopsy showing amounts of histiocytes containing numerous intracellular *Histoplasma spp*. organisms (D. Hematoxylin-Eosin x600; E. Periodic acid–Schiff x1000; F. Grocott-Gomori's methenamine silver stain x1000).

A CT-chest and abdomen revealed a well-circumscribed solitary lesion in the upper lobe of the right lung, with a maximum diameter of 9 mm as well bilateral adrenal lesions, left 6.3 x 6.6 cm and right 6.0 x 4.1 cm, with multiple internal hypodense areas and a small hypodense lesion in Segment III (Figure 3). A CT-guided biopsy of the lung lesion revealed abundant necrosis. Therefore, to exclude malignancy a CT-guided biopsy of the left adrenal lesion was performed.

Histological Findings and Diagnosis of Infection

The histopathology showed an excessive inflammatory reaction with epithelioid histiocytes, singly and in clusters, containing intracytoplasmic inclusions suggestive of microorganisms. A Grocott-Gomori's methenamine silver stain (GMS) revealed abundant small intracellular 3-6 μm microorganisms, which were also PAS positive (Figure 3). On morphological grounds, the yeasts were more compatible with *Histoplasma capsulatum*. PCR analysis confirmed the diagnosis of *Histoplasma capsulatum*. A blood culture drawn after diagnosis was negative for fungi. 

Therapy and Follow-Up

The patient received for 12 months itraconazole 200 mg daily. He was discharged from our hospital two weeks after diagnosis in an improved condition. Sixteen months afterwards, a follow-up of the patient, which was performed on an outpatient basis, revealed complete resolution of the initial symptoms and signs, without any presence of lung or adrenal nodules.

## Discussion

The clinical management of solid renal neoplasms is straightforward. However, there are several infectious as well as non-infectious inflammatory conditions that can mimic malignancy of the urinary system, thus challenging the clinical diagnosis. Although the majority of these processes are rare a high index of suspicion is required in order to reach the diagnosis is important. Moreover, there are for the moment no relevant guidelines on the diagnostic criteria for retroperitoneal infections in clinical practice, even though they can be dangerous and life-threatening [1].

The infection of the kidney or pyelonephritis has an incidence of 15-17 cases per 10,000 women and 3-4 cases per 10,000 men [2]. Most of the cases present with typical features and the diagnosis can be easily documented with urinalysis and clinical symptoms. There are, however, some atypical cases, especially those caused by non-bacterial pathogens, in which the findings may be confusing, making the differential diagnosis from malignancy difficult. Among the various conditions causing the so-called renal pseudotumours are fungal infections (such as *Aspergillus spp.,* *Histoplasma spp.*, *Candida albicans*), xanthogranulomatous pyelonephritis (mostly associated with *Proteus mirabilis *and *Escherichia coli*), renal actinomycosis (*Actinomyces israelli*) and renal mycobacterial infections [2].

Fungal pyelonephritis is especially common in immunocompromised patients such as patients with haematological malignancies, diabetes mellitus, human immunodeficiency virus (HIV), neutropenia, or immunosuppression after organ transplantation [2]. Invasive aspergillosis has been reported in 3.6% to 10.3% of allogeneic HSCT recipients, with higher rates in those with unrelated or HLA-mismatched donors [3]. Neutropenia has traditionally been the most significant risk factor [4]. Other risk factors of invasive aspergillosis are aggressive management of GvHD, older age, use of corticosteroids, and uncontrolled underlying disease [3, 5]. Aspergillosis occurs within a mean period of 78 days after transplantation. However recent trends show an increase in late-onset infections, which now seems to account for most cases of invasive aspergillosis [3, 5], like our case (Case 1) which developed aspergillosis seven months after HSCT. Interestingly, in our case, the first finding was a renal pseudotumour, which retrospectively could be attributed to the onset of aspergillosis. The patient presented with aspergillus pneumonia 10 months after the recognition of the renal lesion and six months after CAR T-cell therapy, which has also been repeatedly associated with invasive fungal infections, and especially *Aspergillus spp* [6]. According to previous reports, renal aspergillosis may be either caused by haematogenous spread, or ascending infection from a urinary tract, or secondary to obstructive uropathy [7]. The former usually involves the renal cortex first and then the renal medulla, the latter two usually occur in the pelvicalyceal system[8]. In our case we observed mainly an involvement of the renal cortex and subsequent involvement of the renal pelvis, supporting the hypothesis of a haematogenous dissemination. 

The most commonly reported symptoms of patients with renal aspergillosis include fever, haematuria, lumbodynia, pyuria, and perineal discomfort, which can all be considered unspecific for the diagnosis [6] However, a subset of patients is asymptomatic. CT and MRI findings are of great importance for the early detection of renal aspergillosis, but vary significantly between patients, frequently being a heterogeneous mass with secondary changes such as abscess formation or necrosis [7, 9]. Other findings, such as vascular occlusion and subsequent renal infarction due to fungal hyphae clumps have been also suggested to be characteristic of renal aspergillosis [9]. The initial delay in establishing the diagnosis usually affects the success rate of treatment, which could be responsible for the fact that in our case the renal tumoral lesion persisted after intravenous anti-fungal treatment. Recent reports suggest that due to the high mortality rate of invasive aspergillosis especially in transplant recipients, a radical or partial nephrectomy, depending on the extent of the disease, could be the main therapeutic strategy followed by adjuvant antifungal treatment [8, 9].

Renal mycobacterial infection is another condition that can appear as a pseudotumour of the kidney, in form of solitary or multiple well-defined parenchymal nodules of variable size [2]. Among mycobacterial infections of the kidney the involvement of *Mycobacterium bovis* after BCG instillation figures prominently. BCG immunotherapy is long known to be associated with localised (such as cystitis, prostatitis, epididymo-orchitis, and pyelonephritis) as well as systemic complications. Life-threatening BCG sepsis has also been reported in only 0.4% of patients [10]. A variety of terms have been used to describe these complications, such as “tuberculosis,” “BCGitis,” “hypersensitivity reaction,” “granulomatous complication,” or simply, “M. bovis infection” [11]. The reported incidence of systemic BCG infection after BCG instillation ranges between 3% and 7% [12]. The onset of these complications can occur from months to years after the last instillation, whereas the longest reported interval between BCG administration and occurrence of a complication (in this case epididymo-orchitis) is 17 years [11]. Renal involvement of *Mycobacterium bovis* after BCG instillation is rare and according to a recent review accounts for 3.5% of the complicated cases [11]. This involvement can either be asymptomatic or present as a granulomatous nephritis and in almost half of the reported cases as a tumour mass. The mechanism of renal pseudotumour formation following intravesical BCG therapy (vesicoureteral reflux vs haematogenous spread) remains controversial [13]. Several parameters have been investigated for their implication in the development of such a complication, such as immunosuppression, time interval between **TUR** and the first BCG instillation, number of BCG instillations, previous operations (prostatectomy or transurethral removal of urethral stone). In this context, recent studies suggest that the odds of developing BCG-related complications likely depend mostly on the host’s characteristics [11]. 

Patients with bladder cancer frequently undergo imaging surveillance of the upper urinary tract and the presence of a renal mass, which as in our case (Case 2) can cause a diagnostic dilemma. Senes et al. suggested a "central unaffected calyx sign" as a specific characteristic imaging feature for BCG infection. This finding was based on the assumption that the mechanism of renal involvement is due to retrograde spread from vesicoureteral reflux compared to malignancies and primary renal tuberculosis, which usually cause destruction of the renal calyces rather than their displacement [13]. Although this finding has not been reproduced in order to be used for diagnostic purposes it was present in our case. Importantly, BCG infection after BCG installation had been missed from the initial differential diagnosis in most of the reported cases in the literature until microbiologic or histological documentation of *Mycobacterium bovis*, as in our case [11]. This phenomenon emphasises the need of maintaining a high index of clinical suspicion based on a previous history of BCG exposure. For cases with a clinical history of treatment with intra-vesicle BCG an invasive procedure and a subsequent histological confirmation can be at least delayed, and follow-up imaging could be used to document possible resolution. Importantly, surgical procedures can be avoided, and a pre-operative fine needle biopsy could help to avoid any complications originating from unnecessary surgical treatment [14].

The infections of the adrenal glands are an important yet relatively unrecognised clinical entity. The adrenal gland can be infected by various pathogens, including a broad spectrum of viruses, fungi, and bacteria. Patients with impaired immune systems are particularly susceptible to either primary adrenal infection or disseminated microbial disease [15]. However, reportedly immunocompetent patients have clinical or pathological evidence of primary adrenal involvement [16]. Moreover, infectious agents are still a major cause of primary adrenal insufficiency/Addison’s disease. The most common bacterial pathogen involving the adrenal glands and causing adrenal destruction is *Mycobacterium tuberculosis*. Fungal infections are relatively uncommon but most reported cases included *Histoplasma spp.* [15]. According to the literature the following infectious pathogens have been reported to cause primary adrenal insufficiency/Addison’s disease: *Mycobacterium tuberculosis, Histoplasma capsulatum, Blastomyves dermatidis, Cryptococcus neoformans, Pneumocystis carinii, Paracoccidioidomycosis, Coccidioides immitis* and *Cytomegalovirus* (CMV) [15]. 

Histoplasmosis usually develops as a pulmonary infection, causing frequently a mass lesion in a sub-pleura location, and can often be indolent or asymptomatic. *Histoplasma spp.* spreads from the lungs to the adrenal glands by haematogenous dissemination and the adrenal glands are the most commonly involved extrapulmonary site in disseminated histoplasmosis [17]. We believe that our patient (Case 3) had an asymptomatic lung histoplasmosis corresponding to the solitary lesion in the upper lobe of the right lung, which disseminated leading to the bilateral adrenal gland and liver involvement. According to the literature, typical adrenal fungal involvement is bilateral, however, unilateral involvement has also been reported [17]. Immunocompromised individuals are at the greatest risk for disseminated histoplasmosis, whereas histoplasmosis occurs in 0.65% of cases among non-AIDS patients who develop fungal infections [18]. Our patient had no history of immunocompromisation, however, was relatively old, which could implicate an age-related immunosenescence, possibly contributing to an increased susceptibility to infection [19]. Primary adrenal insufficiency occurs in 5-71% of adrenal histoplasmosis and has been reported to be the most common cause of death in these patients [18]. Our patient developed some so-called Addisonian symptoms, such as fever, hypotension, hyperkalaemia, hyponatremia, but had a normal ACTH stimulation test, suggesting a mild Addison’s disease. Most patients with histoplasmosis of the adrenal glands have non-specific symptoms, including chronic fatigue syndrome with a great variety of duration of the presenting symptoms. Moreover, they have frequently clinical signs, laboratory findings, and radiological features resembling neoplasms [18]. In this context, the typical imaging finding in adrenal histoplasmosis is adrenal enlargement with frequent areas of necrosis and the differential diagnosis from a neoplasm can be challenging. In most cases, a biopsy is required to reach the diagnosis. Recently, the endoscopic ultrasound-guided fine-needle aspiration has been suggested as a highly effective tool in the diagnosis of adrenal gland inflammation [20]. Importantly, obtaining an adequate patient travel history is of major significance, since it can raise as in our patient the possibility of infection and can facilitate earlier diagnosis.

## Conclusions

In conclusion, our report highlights some examples in which infections of the kidney and the adrenal glands imitate clinically and radiologically neoplasms, complicating differential diagnosis from tumours. Importantly, there are for the moment no relevant guidelines and consensus on the diagnostic criteria for retroperitoneal infections in clinical practice, even though the appearance of infection can be dangerous and life-threatening. However, rare, infectious pseudotumours have to be taken into consideration, in particular, if risk factors, patient’s history, or symptoms support such a diagnosis.
